# Sexual dysfunction and depression in Behçet’s disease in comparison to healthy controls

**DOI:** 10.1007/s00296-021-05000-4

**Published:** 2021-09-28

**Authors:** Sebastian-Jonas Saur, Alexandra Schlögl, Torsten Schmalen, Simon Krittian, Ann-Christin Pecher, Melanie Henes, Theodoros Xenitidis, Jörg Henes

**Affiliations:** 1 Department of Women’s Health, Women’s University Hospital, University Hospital Tuebingen, Tübingen, Germany; 2 University of Tuebingen, School of Medicine, Tübingen, Germany; 3 Department of Dermatology, University Hospital of Tuebingen, Tübingen, Germany; 4 Centre for Interdisciplinary Clinical Immunology, Rheumatology and Auto-Inflammatory Diseases and Department of Internal Medicine II (Oncology, Haematology, Immunology, Rheumatology, Pulmonology), University Hospital Tuebingen, Otfried-Mueller-Strasse 10, Tübingen, Germany; 5 Department of General und Visceral Surgery, Heilig Geist Hospital Köln, Cologne, Germany

**Keywords:** Behçet syndrome, Depression, Sexual dysfunction, Erectile dysfunction, Autoimmune diseases

## Abstract

Behçet’s disease (BD) can affect the genital system and is more common in Middle Eastern countries and Asia but also occurs in Caucasian people. Aim of this study was to evaluate the prevalence of sexual dysfunction (SD) and depression in patients with BD compared to a healthy control group (HCG). In addition, differences with regard to depression and patients’ origin were evaluated. This prospective, monocentric study included 106 consecutive patients from our specialized BD outpatient clinic. Patients were asked to fill out the paper based standardized and validated questionnaires International Index of Erectile Function (IIEF), the Female Sexual Function Index (FSFI) and the Beck Depression Inventory (BDI). In addition, 206 healthy controls were asked to fill out the questionnaires. 106 patients with BD were evaluated and 206 participants in the HCG. The mean age in BD group was 40.5 years as compared to 44.4 years in the HCG. Half of the patients had Middle Eastern and half Caucasian origin. SD was found in 24.5% of all subjects. Only 6.9% of male patients showed signs of SD, while half of the women’s group was suffering from SD. The prevalence for SD was significantly higher in women with Middle Eastern ethnic origin compared to women with Caucasian origin (75 vs. 33.3%, *p* = 0.024). Erectile Dysfunction occurred in 55% of all male patients which was not statistical different from the HCG. Genital ulcers affected 73.6% of all patients. Depression was found in 36.7% of all subjects as compared to 6.25% in the HCG (*p* < 0.001). Both, SD and depression correlated positively in males (*p* = 0.017) and females (*p* = 0.013). SD and depression are very common problems in BD and should be addressed by the treating physician. Both manifestations are intensifying each other. Depression especially is more prevalent compared to the healthy population.

## Introduction

Behçet’s disease (BD) is a systemic vasculitis. The most common manifestations are oral and genital ulcerations but it also affects the eyes and other organs. BD is also called the “Silk Road disease”, because it is more common in countries along the historical silk road, especially Turkey, Israel, Iraq and Iran, may be possibly due to its association with the human leukocyte antigen (HLA) B51. The prevalence in Turkey and Asia is up to 370/100.000. Prevalence in Europe and the USA range between 0.1 and 7.5/100.000. BD is more often found in young men (men vs. woman 3:1) [1, 2].

The definition of sexual dysfunction (SD) includes problems with somatic sexual function (erection, lubrication), desire, arousal, satisfaction, pain and orgasm. To evaluate these problems the International Index of Erectile Function (IIEF) for men and, respectively, the Female Sexual Function Index (FSFI) for women are standardized and validated instruments [3, 4].

Aim of our study was to evaluate the prevalence of SD in patients with BD compared to healthy controls, as well as analysing differences between patients from different origins. In addition, we analysed the prevalence of major depression (MD) and its correlations.

## Methods

This prospective, monocentric study included 106 patients with BD from our specialized outpatient clinic. The study was approved by the ethics committee of the University Hospital Tuebingen (264/2015BO2) and all patients gave written informed consent. Data on clinical symptoms, pathergy phenomenon and HLA-B51 was compiled. The Healthy Control Group (HCG) is composed of 87 women and 119 men.

The IIEF and FSFI were used for assessing SD and the Beck Depression Inventory (BDI) [5] was used for psychiatric assessment. The IIEF does not include any pain domain. In consequence we added questions about pain during and after sexual intercourse in men. Within the IIEF there is a differentiation between SD, which refers to the whole questionnaire and erectile dysfunction (ED), which only refers to the domain of erectile function.

For statistics we used SPSS to analyze the prevalence of SD, MD and clinical symptoms. An independent *t* test was used to analyze differences in subjects with Middle Eastern ethnic and Caucasian origin as well as in gender categories. A low score in FSFI (cut off ≤ 26.55 points) and IIEF (cut off ≤ 21 points) meant a higher occurrence of SD, while higher scores in BDI (cut off ≥ 14 points) indicated a depression. The cutoff for assessing the severity of depression are 0–13 points for no depression, 14–19 for mild depression, 20–28 for moderate depression and 29–63 for severe depression. We also used a Chi squared test to show differences in nominal variables, such as gender and ethnicity. A *p* value ≤ 0.05 was judged as significant.

## Results

One hundred and six patients with BD were included in this study. Half of the group had a Middle Eastern ethnic origin and 50% were Caucasians, with 56.6% being women and 43.4% men. The HCG was composed of 87 women with a median age of 34 years (ranging from 18 to 64) and 119 men with a median age of 52 years (ranging from 20 to 69). The median age of patients was 40.5 years (range 18–64 years) and 83% lived in a relationship. Every patient had oral aphthosis, while 73.6% suffered from genital aphthosis. Other manifestations and symptoms are summarized in Table 1 and significant differences between women and men are highlighted. Joint involvement was significantly more frequent in women than in men (71.7 vs. 45%; *p* = 0.006) and eye involvement was significantly more frequent in men than in women (63.3 vs. 30.4%; *p* = 0.001). More than half (55.7%) of all patients were HLA-B51 positive, men more often than women (70.2 vs. 44.2%, *p* = 0.009).

**Table 1:** Frequency of type Behcet manifestations in our cohort showing some clinical significant differences (bold) between men and women; abbreviations: *n.s.* not significant, *n.d.* not done

Symptoms	Frequency All	Frequency Men	Frequency Women	*p*
Oral aphthosis	100%	100%	100%	n.s
Genital aphthosis	**73.6%**	**61.7%**	**89.1%**	**0.001**
Skin involvement total	84%	85%	82.6%	0.739
Papulopustulosis	45.3%	n.d	n.d	n.d
Erythema nodasum	8.5%	n.d	n.d	n.d
Eye involvement	**49.1%**	**63.3%**	**30.4%**	**0.001**
Joint involvement total	**56.7%**	**45%**	**71.7%**	**0.006**
Arthralgia	66.6%	n.d	n.d	n.d
Arthritis	28.3%	n.d	n.d	n.d
Pathergy positive	29.2%	n.d	n.d	n.d
HLA B51 positive	**55.7%**	**70.2%**	**44.2%**	**0.0009**
Gastrointestinal involvement	22.2%	24.7%	20.1%	n.s

Altogether, SD was found in 50% of female patients with BD compared to 44.8% in the HCG. 36.4% of patients indicated BD as a reason for not having sexual intercourse. Only 6.9% of the male patient group showed signs of SD according to the IIEF, ED was found in 55% (mild 36.7%, moderate 3.3% and severe 11.7%) of patients, whereas only 33.3% (mild 21%, moderate 3.4% and severe 8.4%) of the HCG, which was not statistically significant. The prevalence of SD was significantly higher in women with Middle Eastern ethnic origin compared to Caucasian women (75 vs. 33.3%, *p* = 0.024). Scores in FSFI were significantly lower in women with Middle Eastern ethnic origin (19.4 ± 9.03 points) than in women with Caucasian origin (27.2 ± 5.14; *p* = 0.005).

Almost half of the women (43.9%) were suffering from pain during and 36.5% from pain after vaginal penetration. Male subjects also reported pain during (20.4%) and after (18.7%) sexual intercourse. Male and female patients with SD more often had genital aphthosis than patients without SD (87.5 vs. 66.2%). This indicates a significant positive correlation between SD and genital aphthosis (*p* = 0.045).

**Fig. 1:**
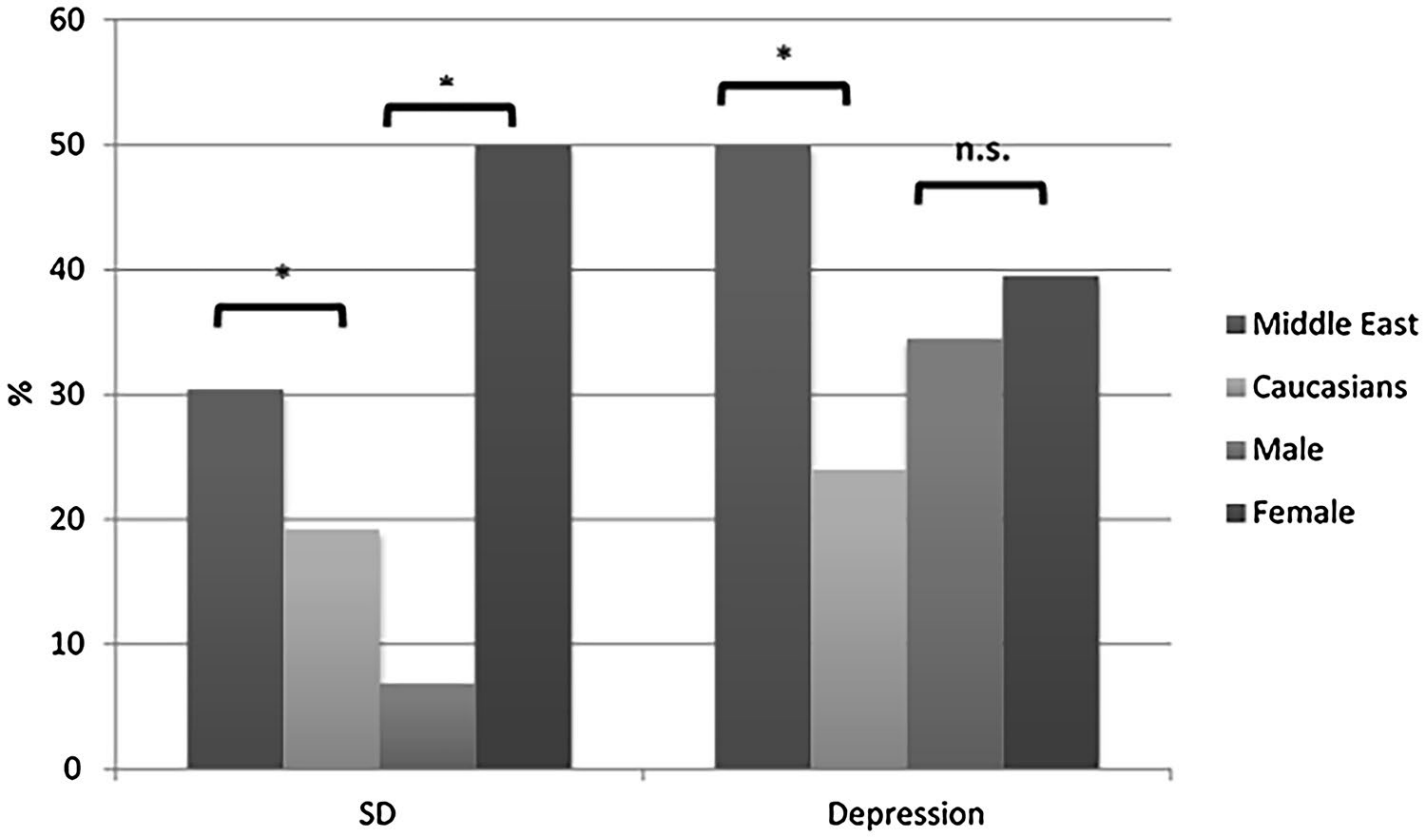
Prevalence of sexual dysfunction (SD) and depression in Behcet patients showing differences in gender and origin; * = Significant, *n.s.* not significant

Depression was found in 36.7% of all subjects (10% mild, 13.3% moderate, 13.3% severe depression) with a non-significant difference between men and women (*p* = 0.611) but with a highly significant difference (*p* = 0.001) compared to the HCG with only 6.25% (5.2% mild, 0.5% moderate and 0.5% severe) Half of the subjects with Middle Eastern ethnic origin showed signs of depression, while only 24% of subjects with Caucasian origin did (Fig. 1).

**Fig. 2:**
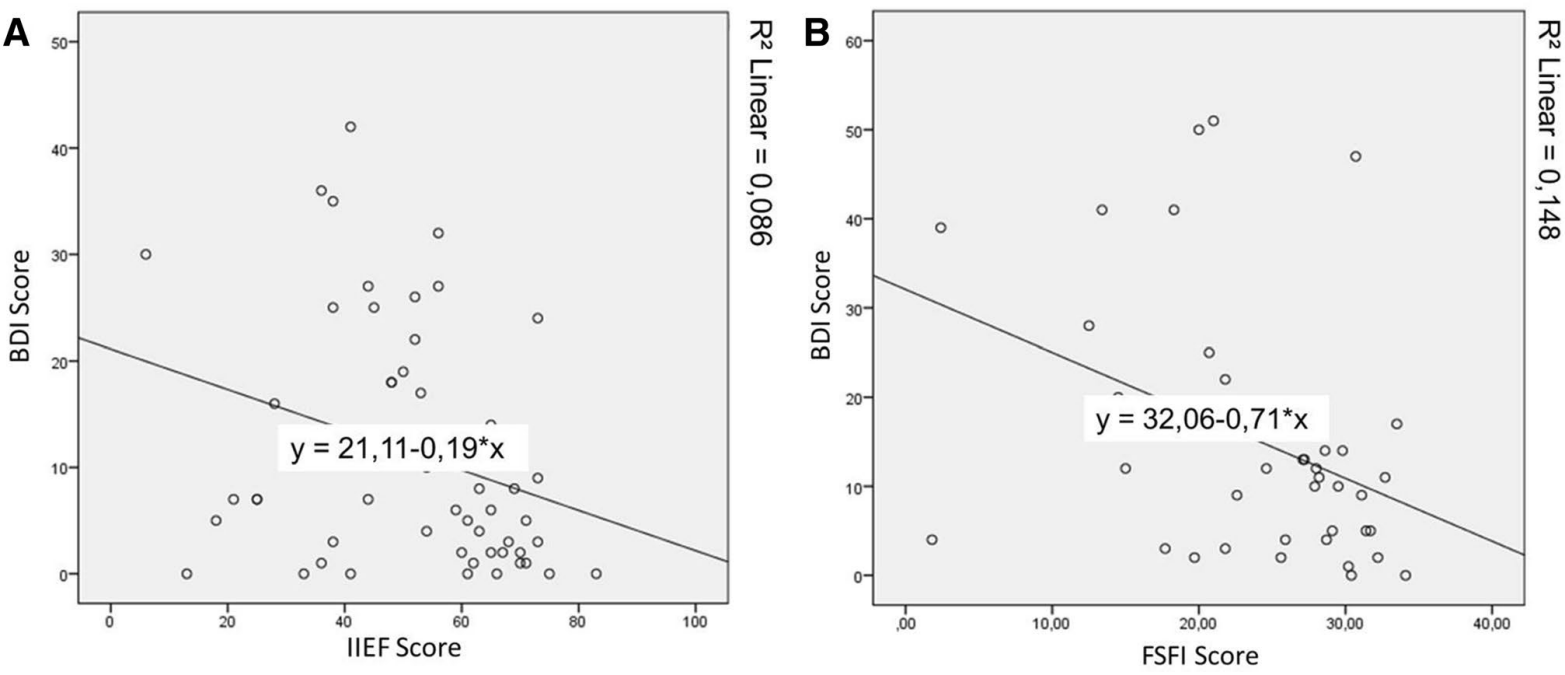
**A** Scatterplot with correlation of scores in BDI and IIEF of male MB patient. **B** Scatterplot with correlation of scores in BDI and FSFI of female MB patient

SD was found in 34.4% of all depressive patients. Both SD and depression correlated positively in male (*p* = 0.017) and female patients (*p* = 0.013). The scores for males and female in BDI and IIEF and BDI and FSFI, respectively, correlated negatively (Spearman-Rho = − 0.327, *p* = 0.017; Spearman-Rho = -0.395, *p* = 0.013) (Fig. 2). This indicates that both depression and SD more often occurred in combination.

Surprisingly more than one third (35.5%, male 35% vs. female 26.1%) of patients with BD stated that frequent sexual intercourse decreased the occurrence of genital aphthosis, while only 7.5% (male 5% vs. female 8.7%) stated, that genital aphthosis occurred more often. Frequent sexual intercourse led to less occurrence of pain during sexual intercourse in 39.6%.

## Discussion

This is the first study on SD in patients with BD in Germany and the first to investigate differences regarding the ethnic background and in comparison to a HCG. We observed that SD is common in patients with BD. Women especially suffered from SD with a similar extent to the women in the HCG (50 vs. 44.8%). This observation is consistent with data from other studies on the prevalence of SD in women in the general population ranging from 42 to 43% [6, 7]. SD was primarily observed in women from Middle Eastern countries (75%) and was more common than in the general population of these countries, ranging from 17.5% to 46.9% [8–10]. Caucasian women with BD tended to have a lower prevalence of SD than women in the HCG. Pain during penetration occurred more frequently than in healthy subjects (8–21%) from other epidemiologic studies [10, 11]. We also found higher rates of pain during vaginal penetration than Kocak et al. in a comparable study with 71 Turkish women with BD [9]. Overall women with BD were affected by SD, pain during vaginal penetration and lubrication troubles more often than comparable healthy subjects and had higher rates of SD than subjects with other rheumatologic diseases [9, 10]. Interestingly, our patients stated that frequent sexual intercourse had a positive influence on the occurrence of genital aphthosis, pain during and after sexual intercourse, and general well-being, which might be a helpful point when counselling BD patients. Consistent with the findings of Yetkin et al., mucocutaneous disease manifestations negatively affect sexual life and promote depression [12]. The same group also noted a testosterone deficiency in the patients with BD, which could contribute to the development of SD. Unfortunately, no data are available from our cohort in this regard.

The overall mean prevalence of ED ranges from 14 to 48% with a higher prevalence found in the United States and in Southeast Asia when compared with Europe [6].

The overall prevalence rates for ED in the German (19.2%) as well as the general European population (30%) are somewhat higher than our results for the HCG [7]. We found a slightly lower prevalence of ED in our HCG (16%). Hiz et al., who also used as well the IIEF domain of erectile function and BDI for depression, found isolated ED more frequently than we did (90 vs. 55%) [13]. Nevertheless, both results are corresponding. Erectile dysfunction appears to be common (63%), particularly in patients with neuro-BD, although our study did not include any patients with neuro-BD [14]. In addition, Hiz et al. described a significant correlation between ED and depression, as we did. Yildiz et al. identified the frequent occurrence of psychological problems in patients with BD as another factor influencing sexual function, whereas other disease manifestations did not seem to have a relevant influence [15]. We could also show that depression in patients with BD is more common than in subjects of our HCG (36.7 vs. 6.25%). In addition, patients with BD from Middle Eastern countries had much higher rates of depression than healthy subjects from the same countries (4.1–8.2%) [16, 17], indicating that not the ethnic origin but the disease influences SD and depression. The rate of depression in patients with Middle-Eastern ethnic origin was higher than in patients with Caucasian origin (*p* = 0.034). Kocak et al. and Yetkin et al. also described a significant correlation between depression and female sexual dysfunction (*p* = 0.013) [9]. These results support the hypothesis that BD has a negative influence on patient’s psychological well-being and indicates that depression and SD influence each other in their intensity. These observations are consistent with the results of a meta-analysis by Talarico et al. [18]

Assessing SD and depression is challenging as you have to convince all patients to fill out a very intimate, multi-sided questionnaire on their sexual behavior, desire and arousal. Self-report may be exaggerated but also understated as respondents may be too embarrassed to disclose private details. In addition, there are several external factors that cannot be influenced and that may contribute to a bias in the results, such as social desirability [19]. Therefore, a strong confidence between patient and physician is necessary to get high quality data. In summary, however, a bias must be assumed for each survey questionnaire, which should be taken into account when interpreting the data [20]. Another problem is that data show that the topic of sexuality is often not addressed in consultations, because the attending physicians feel uncomfortable and are usually not trained in the topic [21]. The relatively small number of 106 patients and the fact that this is a monocentric study limits the significance of this work, but was strengthened by the large healthy comparison group. However, a further comparison of the patient group with the HCG was not possible, because no information on ethnicity was available. SD and depression are influenced by multiple factors. Especially cultural differences play an important role in this context and were not addressed by this study. Nevertheless, our study is the first to evaluate data about SD and depression in BD in Germany and it is the first to differ between patients with Middle Eastern ethnic and Caucasian origin.

## Conclusion

In conclusion we could demonstrate that SD and depression are common problems in patients with BD and that both influence each other. Especially in women, SD is more prevalent than in the healthy population. In consequence we suggest that there should be an assessment of sexual function and psychological state in patients with BD. It should be emphasised in this work that for the first time a larger collective of patients with BD of Caucasian and Middle Eastern descent could be compared, whereby clear differences could be found. A continuing education program for physicians on sexuality and depression would be helpful to improve patient care. We recommend further investigations to gain more epidemiological and clinical data on SD and depression and thus improve the care for our BD patient’s.

## Data Availability

Raw data available on demand.

## References

[1] Zouboulis CC (1999). Epidemiology of Adamantiades-Behcet’s disease. Ann Med Interne (Paris).

[2] Altenburg A, Papoutsis N, Orawa H, Martus P, Krause L, Zouboulis CC (2006). Epidemiology and clinical manifestations of Adamantiades-Behcet disease in Germany—current pathogenetic concepts and therapeutic possibilities. J Dtsch Dermatol Ges.

[3] Rosen RC, Riley A, Wagner G, Osterloh IH, Kirkpatrick J, Mishra A (1997). The international index of erectile function (IIEF): a multidimensional scale for assessment of erectile dysfunction. Urology.

[4] Rosen R, Brown C, Heiman J, Leiblum S, Meston C, Shabsigh R, Ferguson D, D'Agostino R (2000). The female sexual function index (FSFI): a multidimensional self-report instrument for the assessment of female sexual function. J Sex Marital Ther.

[5] Beck AT, Ward CH, Mendelson M, Mock J, Erbaugh J (1961). An inventory for measuring depression. Arch Gen Psychiatry.

[6] Rosen R, Altwein J, Boyle P, Kirby RS, Lukacs B, Meuleman E, O'Leary MP, Puppo P, Robertson C, Giuliano F (2003). Lower urinary tract symptoms and male sexual dysfunction: the multinational survey of the aging male (MSAM-7). Eur Urol.

[7] Corona G, Lee DM, Forti G, O'Connor DB, Maggi M, O'Neill TW, Pendleton N, Bartfai G, Boonen S, Casanueva FF (2010). Age-related changes in general and sexual health in middle-aged and older men: results from the European male ageing study (EMAS). J Sex Med.

[8] Cayan S, Akbay E, Bozlu M, Canpolat B, Acar D, Ulusoy E (2004). The prevalence of female sexual dysfunction and potential risk factors that may impair sexual function in Turkish women. Urol Int.

[9] Kocak M, Basar MM, Vahapoglu G, Mert HC, Gungor S (2009). The effect of Behcet’s disease on sexual function and psychiatric status of premenopausal women. J Sex Med.

[10] Read S, King M, Watson J (1997). Sexual dysfunction in primary medical care: prevalence, characteristics and detection by the general practitioner. J Public Health Med.

[11] Laumann EO, Paik A, Rosen RC (1999). Sexual dysfunction in the United States: prevalence and predictors. JAMA.

[12] Yetkin DO, Celik O, Hatemi G, Kadioglu P (2013). Sexual dysfunction and depression in premenopausal women with mucocutaneous Behcet’s disease. Int J Rheum Dis.

[13] Hiz O, Ediz L, Gulcu E, Tekeoglu I (2011). Effects of Behcet's disease on sexual function and psychological status of male patients. J Sex Med.

[14] Erdogru T, Kocak T, Serdaroglu P, Kadioglu A, Tellaloglu S (1999). Evaluation and therapeutic approaches of voiding and erectile dysfunction in neurological Behcet’s syndrome. J Urol.

[15] Yildiz M, Batmaz I, Sula B, Ucmak D, Sariyildiz MA, Daggulli M, Turkcu F, Karakoc M (2016). Sexual dysfunction in male patients with Behcet’s disease. Acta Reumatol Port.

[16] Topuzoglu A, Binbay T, Ulas H, Elbi H, Tanik FA, Zagli N, Alptekin K (2015). The epidemiology of major depressive disorder and subthreshold depression in Izmir, Turkey: prevalence, socioeconomic differences, impairment and help-seeking. J Affect Disord.

[17] Sadeghirad B, Haghdoost AA, Amin-Esmaeili M, Ananloo ES, Ghaeli P, Rahimi-Movaghar A, Talebian E, Pourkhandani A, Noorbala AA, Barooti E (2010). Epidemiology of major depressive disorder in Iran: a systematic review and meta-analysis. Int J Prev Med.

[18] Talarico R, Elefante E, Parma A, Taponeco F, Simoncini T, Mosca M (2020). Sexual dysfunction in Behcet’s syndrome. Rheumatol Int.

[19] Rosenman R, Tennekoon V, Hill LG (2011). Measuring bias in self-reported data. Int J Behav Healthc Res.

[20] Gaur PS, Zimba O, Agarwal V, Gupta L (2020). Reporting survey based studies—a primer for authors. J Korean Med Sci.

[21] Nahata L, Ziniel SI, Garvey KC, Yu RN, Cohen LE (2017). Fertility and sexual function: a gap in training in pediatric endocrinology. J Pediatr Endocrinol Metab.

